# Individual Learning Needs of Japanese Public Health Dietitians by Years of Experience in Health Promotion

**DOI:** 10.3390/healthcare11121765

**Published:** 2023-06-15

**Authors:** Osamu Kushida, Ayaka Iida, Yusuke Arai, Tatsuya Koyama, Kazumi Tanaka, Ayumi Morooka, Sumie Isobe, Rie Okamoto, Katsushi Yoshita

**Affiliations:** 1 School of Food and Nutritional Sciences, University of Shizuoka, Shizuoka 422-8526, Shizuoka, Japan; 2 Faculty of Health and Social Services, Kanagawa University of Human Services, Yokosuka 238-8522, Kanagawa, Japan; 3 Department of Nutrition, Chiba Prefectural University of Health Sciences, Chiba 261-0014, Chiba, Japan; 4 Faculty of Human Life Sciences, Mimasaka University, Tsuyama 708-8511, Okayama, Japan; 5 Planning Division, Hyogo Prefecture, Kobe 650-8567, Hyogo, Japan; 6 Minami-Uonuma Health Center, Niigata Prefecture, Minamiuonuma 949-6680, Niigata, Japan; 7 Faculty of Health Sciences, Kanazawa University, Kanazawa 920-1192, Ishikawa, Japan; 8 Graduate School of Human Life and Ecology, Osaka Metropolitan University, Osaka 558-8585, Osaka, Japan

**Keywords:** dietitians, administration, public health nutrition, training program, learning needs

## Abstract

Lifelong education for dietitians in Japan is based mainly on competencies according to years of experience. Because learning content differs depending on the desired position and specialty, training programs that reflect the individual learning needs of public health dietitians are needed. This study aimed to assess the individual learning needs of public health dietitians via years of experience in health promotion. In 2021, an online survey of public health dietitians involved in health promotion in prefectures, designated cities, and other municipalities throughout Japan was conducted. Years of experience in health promotion were categorized as early (<10 years), mid-career (10–19 years), and leadership (≥20 years) periods. To ascertain individual learning needs, the survey asked about respondents’ desired final position, career path, and skills they felt they needed to improve in the future. Of the 1649 public health dietitians analyzed, all administrative categories preferred to work as public health generalists in mid-career or leadership periods rather than in the early period. In municipalities, more public health dietitians across all experience categories selected “professional competence”, such as knowledge in specialized areas and nutritional guidance techniques. It was suggested that public health dietitians in the mid-career and leadership periods have individual learning needs, in both nutrition specialists and public health generalists.

## 1. Introduction

The Japan Dietetic Association’s continuing education system has basic and advanced education divisions for various professional fields, including health, medical care, and welfare, and issues certifications, in eight areas, including public health nutrition, based on credits gained and skill achievements [1]. The continuing education system also categorizes competencies according to years of experience and promotes continuous professional development according to achievements for each year of experience [2]. The Public Health Division of the Japan Dietetic Association also conducts group activities to examine the following competencies of public health dietitians according to years of experience during training sessions: practical ability, organizational role and ability to perform, and educational and research capability [3].

Lifelong professional development for healthcare professionals requires a balance between group and individual learning needs. This is because addressing the learning needs of the group may not always adequately address the needs and interests of individual group members [4]. A previous study of Japanese clinical nurses identified individual learning needs such as “theory, knowledge, technique, and attitude necessary for nursing practice to meet the specialty of each nursing unit” and “theory, knowledge, technique, and attitude of interpersonal relationships and communications necessary for nursing practice and nursing task performance” [5]. Based on this, educational programs have been developed to reflect both group learning needs, including the skills necessary for cultivating the desired professional image, and individual learning needs, including the skills that each nurse wants to learn [6]. However, no studies have examined the individual learning needs of public health dietitians in Japan.

Public health dietitians need to acquire different skills depending on their desired position and specialty. Clarifying individual learning needs in addition to the previously examined group learning needs of public health dietitians will aid in the planning of tailor-made training programs. Therefore, this study aimed to assess individual learning needs by years of experience in health promotion in order to consider a training program for public health dietitians.

## 2. Materials and Methods

### 2.1. Study Design and Participants

A cross-sectional study was conducted from January to March 2021, using an online survey of public health dietitians nationwide who worked in prefectures, designated cities (cities), and other municipalities (municipalities). Because public health dietitians in Japan are sometimes assigned to municipal hospitals or boards of education, the participants in this study were limited to those in charge of health promotion. In this study, health promotion was defined as “activities aimed at promoting community health and improving nutrition carried out by the departments in charge of health (head office) or public health centers” for prefectures and cities and “activities aimed at promoting community health, including maternal and child health as well as adult health, such as specific health checkups and health guidance” for municipalities. Total work history was defined as the number of years of experience in health promotion, and three categories were established based on a previous study that investigated the association between competencies and related factors such as years of experience in public health nurses [7]: early (<10 years), mid-career (10–19 years), and leadership (≥20 years).

The eligibility criteria were as follows: participants had indicated in a Ministry of Health, Labour and Welfare survey that they were public health dietitians; their employment status was full-time or near full-time (i.e., working at least 4 days a week for at least 6 h a day); and they had experience in health promotion work. Exclusion criteria were that they were primarily in charge of areas other than health promotion (e.g., welfare, childcare, elderly care, school boards, and medical care) at the time of the survey.

### 2.2. Setting

A letter requesting participation in the survey was mailed in January 2021 to public health dietitians working in departments in charge of health in prefectures, cities, and municipalities throughout Japan. The cover letter included a URL and QR code to access the online survey, which described the purpose of the survey and stated that the survey was anonymous, participation was voluntary, informed consent would be indicated by responding to the questionnaire, and there would be no disadvantage if they did not respond. If they were unable to respond online, they could download the survey as a PDF from a special website provided by the research group and return it by fax or e-mail. Furthermore, the authors asked the Japanese Association of Public Health Center Registered Dietitian and the Public Health Division of the Japan Dietetic Association to publicize the survey to their members. The response period was from 29 January 2021 to 2 March 2021. To ensure the anonymity of the respondents, all responses were collected by a survey company. The research protocol was approved by the Research Ethics Committee of the Graduate School of Life Sciences, Osaka City University (application numbers 20–35 and 20–39; approved 11 November 2020 and 9 December 2020).

### 2.3. Measurements

In a previous study of Japanese public health nurses, 66% of participants did not want to be empowered to lead and manage staff [8]. Public health dietitians in Japan likely also include those who want to be promoted in the future and those who want to continue working in their current positions. Therefore, in the present study of individual learning needs, the questionnaire began by asking the respondent’s desired final position as follows: officer, supervisor, or management position.

The knowledge and skills needed by public health dietitians mainly include those in the areas of public health and public health nutrition [9,10]. Therefore, the respondents were asked to indicate their intended career path from a list of 12 items, including “I want to continue working as a nutrition specialist”, “I want to be promoted and work as a nutrition specialist”, “I want to continue working as a public health generalist”, and “I want to be promoted and work as a public health generalist”.

In a previous study investigating the learning needs of registered nurses, participants were asked about the knowledge and skills they felt they needed to improve in their area of specialty practice [11]. At the end of the survey, 10 questions were developed regarding skills that the respondents felt they needed to improve in the future. The questions developed by the authors (8 registered dietitians and 1 public health nurse who work for government agencies or universities) were confirmed to cover the learning needs of public health dietitians in the areas by stakeholders (2 registered dietitians and 1 physician who work for ministries or government agencies). Each item was considered valid because similar competencies were reported in previous studies on Japanese dietitians or public health dietitians as follows [3,12,13,14,15,16,17]: (1) administrative competence [12], (2) professional competence [13], (3) community support capacity [14], (4) research competence [3], (5) coordination competence [15], (6) policy-making competence [16], (7) information dissemination competence [15], (8) organizational management competence [3], (9) risk management competence [17], and (10) other. In the questionnaire, several examples were included as definitions for each item. Respondents were asked to respond to up to three items, regardless of rank, that they felt were particularly applicable regarding their career path and skills they felt they needed to improve in the future.

The questionnaire also asked about respondents’ demographics, including gender, age, educational background, and current position. Certification as a registered dietitian/dietitian in Japan requires completion of a registered dietitian/dietitian training course at a university, junior college, or vocational school [18]. Therefore, those institutions and graduate schools were provided as response options for educational background.

### 2.4. Statistical Analysis

Statistical analysis was conducted for each administrative category: prefectures, cities, and municipalities. Because only three respondents were in management positions at the prefectural level at the time of the survey, the supervisory and management positions were combined into a single category in the results of both current and desired final positions. To examine individual learning needs, a binary logistic regression analysis was performed. The desired final position, intended career path, and skills that dietitians felt they needed to improve in the future were the dependent variables, and years of experience in health promotion was the independent variable. In addition to the number of respondents to which each item was applied, odds ratios (ORs) and 95% confidence intervals (95% CIs) were calculated for summary statistics. IBM SPSS Statistics 27 (IBM Japan, Ltd., Tokyo, Japan) was used for all analyses. The significance level was set at *p* < 0.05 (two-sided test).

## 3. Results

Of the 1806 respondents, 1649 were included in the analysis after excluding those who were not qualified as dietitians (prefectures (*n* = 5), cities (*n* = 7), municipalities (*n* = 4)), those who had 0 years of experience in health promotion (prefectures (*n* = 5), cities (*n* = 6), municipalities (*n* = 128)), and those with missing items for analysis (prefectures (*n* = 1), cities (*n* = 1)). Overall, 1594 (96.7%) of the respondents were women, 394 (23.9%) were in their 20s, 439 (26.6%) were in their 30 s, 458 (27.8%) were in their 40s, 358 (21.7%) were 50 or older, and 1145 (69.4%) had a bachelor’s degree or higher (Table 1).

Regarding their current position, 1407 (85.3%) respondents were officers. As for respondents who indicated “supervisory/management” by years of experience, 43 (4.4%) were in the early period (1 (0.5%) in prefectures, 14 (8.0%) in cities, and 28 (4.7%) in municipalities), 88 (22.6%) were in the mid-career period (20 (16.0%) in prefectures, 20 (24.4%) in cities, and 48 (26.2%) in municipalities), and 111 (39.2%) were in the leadership period (32 (28.1%) in prefectures, 20 (37.7%) in cities, and 59 (50.9%) in municipalities).

Of the individual learning needs, the following six items were selected as the intended career path in ≥10% of the responses: “I want to continue working as a nutrition specialist”, “I want to be promoted and work as a nutrition specialist”, “I want to continue working as a public health generalist”, “I want to be promoted and work as a public health generalist”, “I want to transfer to a department outside of health promotion services (in a bureau)”, and “I want to change jobs outside of government (non-education/research)”. In terms of skills that the respondents felt they needed to improve in the future, nine items, except for “other”, were selected in ≥10% of the responses.

### 3.1. Individual Learning Needs in Prefectures

There were no significant differences in the number of respondents who indicated their desired final position as “supervisory/management” in both the mid-career and leadership periods compared with the early period (OR 0.98; 95% CI 0.63–1.53 and OR 1.16; 95% CI 0.73–1.84, respectively) (Table 2).

Regarding their career path, most respondents selected “I want to continue working as a nutrition specialist” regardless of years of experience: 100 (49.5%) in the early period, 48 (38.4%) in the mid-career period, and 44 (38.4%) in the leadership period. In a comparison among years of experience, more respondents selected “I want to continue working as a public health generalist” in the leadership period than in the early period (OR 2.61; 95% CI 1.56–4.39).

Regarding the skills they felt they needed to improve in the future, “professional competence” was highest in the early period, at 114 (56.4%), while “coordination competence” was highest in the mid-career period, at 61 (48.8%), and in the leadership period, at 62 (54.4%). In a comparison among years of experience, more respondents selected “risk management competence” in the mid-career period than in the early period (OR 1.97; 95% CI 1.18–3.29). Compared with the early period, more respondents in the leadership period selected “coordination competence” (OR 1.78; 95% CI 1.12–2.83), while fewer selected “community support capacity” (OR 0.58; 95% CI 0.34–0.99). Compared with the early period, more respondents in both the mid-career and leadership periods selected “organizational management competence” (OR 3.94; 95% CI 2.22–6.97 and OR 3.31; 95% CI 1.83–5.97), while fewer selected “professional competence” (OR 0. 24; 95% CI 0.15–0.40 and OR 0.22; 95% CI 0.13–0.37).

### 3.2. Individual Learning Needs in Cities

There were no significant differences in the number of respondents who indicated their desired final position as “supervisory/management” in both the mid-career and leadership periods compared with the early period (OR 1.09; 95% CI 0.65–1.85 and OR 1.39; 95% CI 0.75–2.57, respectively) (Table 3).

Regarding their career path, most respondents selected “I want to continue working as a nutrition specialist” regardless of years of experience: 107 (61.5%) in the early period, 51 (62.2%) in the mid-career period, and 27 (50.9%) in the leadership period. In a comparison of years of experience, more respondents selected “I want to be promoted and work as a public health generalist” in the mid-career period than in the early period (OR 2.35; 95% CI 1.06–5.20).

Regarding the skills they felt they needed to improve in the future, “professional competence” was the highest, at 123 (70.7%) in the early period, “information dissemination competence”, at 37 (45.1%) in the mid-career period, and “professional competence”, “coordination competence”, and “policy-making competence”, at 21 (39.6%) in the leadership period. In a comparison among years of experience, more respondents selected “information dissemination competence” in the mid-career period than in the early period (OR 2.10; 95% CI 1.21–3.62). Compared with the early period, fewer respondents in both the mid-career and leadership periods selected “professional competence” (OR 0.25; 95% CI 0.14–0.44 and OR 0.27; 95% CI 0.14–0.52).

### 3.3. Individual Learning Needs in Municipalities

More respondents indicated their desired final position as “supervisory/management” in both the mid-career and leadership periods compared to the early period (OR 1.63; 95% CI 1.17–2.27 and OR 2.81; 95% CI 1.84–4.30, respectively) (Table 4).

Regarding their career path, most respondents selected “I want to continue working as a nutrition specialist” regardless of years of experience: 397 (66.2%) in the early period, 129 (70.5%) in the mid-career period, and 82 (70.7%) in the leadership period. In a comparison among years of experience, more respondents selected “I want to be promoted and work as a public health generalist” in the mid-career period than in the early period (OR 1.82; 95% CI 1.08–3.07). Compared with the early period, more respondents in the leadership period selected “I want to continue working as a public health generalist” (OR 2.80; 95% CI 1.78–4.40), while fewer selected “I want to be promoted and work as a nutrition specialist” (OR 0.52; 95% CI 0.28–0.96).

Regarding the skills they felt they needed to improve in the future, most respondents selected “professional competence” regardless of years of experience: 475 (79.2%) in the early period, 103 (56.3%) in the mid-career period, and 64 (55.2%) in the leadership period. In a comparison between years of experience, more respondents selected “policy-making competence” in the mid-career period than in the early period (OR 1.99; 95% CI 1.40–2.83), while fewer selected “community support capacity” (OR 0.61; 95% CI 0.43 –0.86). Compared with the early period, more respondents in both the mid-career and leadership periods selected “organizational management competence” (OR 1.93; 95% CI 1.25–2.96 and OR 2.59; 95% CI 1.61–4.18), while fewer selected “professional competence” (OR 0.34; 95% CI 0.24–0.48 and OR 0.32; 95% CI 0.21–0.49).

## 4. Discussion

In each administrative category (i.e., prefectures, cities, and municipalities), more municipal public health dietitians indicated their desired final position as “supervisory/management” in both the mid-career and leadership periods than in the early period. There may be more public health dietitians in the early period in municipalities who do not wish to be promoted compared with other administrative categories. A previous study of public health nurses who had experience as clinical instructors for nursing students suggested that professional identity and self-confidence are associated with self-evaluation as a clinical instructor [19]. In terms of the association between job satisfaction and demographics among registered dietitians/dietitians in Japan, there were no significant differences according to gender, but younger respondents had lower job satisfaction [20]. Therefore, further research is needed to clarify factors that are associated with the desire for promotion, such as self-confidence or job satisfaction. Furthermore, it will be necessary to examine training programs that reflect the individual learning needs of those who do not want to be promoted.

Regarding career paths, more public health dietitians in all administrative categories wanted to work as public health generalists in mid-career or leadership periods than in the early period. Meanwhile, more municipal public health dietitians wanted to work as nutrition specialists across all experience categories. Since the enactment of the Community Health Law in Japan, the role of interpersonal services has been assigned to municipalities [21]. Because municipal public health dietitians in mid-career and leadership periods play a role as public health generalists as well as public nutrition specialists, which includes providing nutritional guidance, educational programs need to be developed that reflect both learning needs.

With respect to skills they felt they needed to improve in the future, a previous study examining the competencies of Japanese public health dietitians according to years of experience also showed skills such as “collaboration with other institutions” in the mid-career stage (≥10 years) and “policy management capabilities” in the management stage (≥15 years) [3]. Furthermore, another previous study that identified a consensus on competencies for public health nutrition workforce in Europe according to work category also indicated that manager-level workers require advanced-level competency in leadership and strategic planning [22]. Therefore, the results suggest that the group and individual learning needs of public health dietitians according to years of experience are similar. However, among municipal public health dietitians across all experience categories, more selected “professional competence” such as knowledge in specialized areas and nutritional guidance techniques. A previous study of operating room nurses also showed a strong need for practical education in “management of surgical equipment” across all years of clinical experience [23]. Taken together, these findings suggest that training programs are also needed for the main domain in the profession, regardless of years of experience.

There are several limitations to this study. First, because individual learning needs are assessed using a binary scale of applicable and not applicable, the strength of the need is not clear. Many previous studies have used a 5-point Likert scale, so future studies should also assess the strength of individual learning needs. Second, the validity and reliability are not clear for the skills that participants felt they needed to improve in the future. However, the survey should have content validity, given that similar competencies were reported in previous studies [3,12,13,14,15,16,17], and the items were created with key stakeholders. Research on learning needs assessments is limited; however, a previous study also developed an assessment questionnaire using a literature review and key stakeholders as sources [11]. In the development of learning needs assessment tests, it will also be necessary to examine construct validity in the future. Third, because the number of public health dietitians approached by each administration to take the survey is not known, the response rate cannot be calculated. A low response rate would introduce the risk of selection bias among those interested in developing a training program, which is the overall objective of the research.

The main strength of this study is that the survey was conducted in all levels administrative categories nationwide in Japan and included participants regardless of the current position. Because group learning needs of public health dietitians are similar in Japan and Europe [3,22], the individual learning needs clarified in this study are possibly also similar to those in other countries.

## 5. Conclusions

The findings of this study, which examines individual learning needs according to years of experience in health promotion among public health dietitians, suggest that those in the early period have a particular need to learn to become nutrition specialists, while those in the mid-career and leadership periods have a particular need to learn to become public health generalists. Furthermore, there was a strong need for professional competence across all experience categories in municipalities, suggesting the possibility of individual learning needs for both nutrition specialists and public health generalists in the mid-career and leadership periods.

## Figures and Tables

**Table 1:** Demographics and current position of participants.

	Total (*n* = 1649)	Prefectures (*n* = 441)	Cities ^1^ (*n* = 309)	Municipalities ^2^ (*n* = 899)
Gender				
Women	1594 (96.7)	421 (95.5)	297 (96.1)	876 (97.4)
Men	55 (3.3)	20 (4.5)	12 (3.9)	23 (2.6)
Age				
20s	394 (23.9)	109 (24.7)	65 (21.0)	220 (24.5)
30s	439 (26.6)	75 (17.0)	85 (27.5)	279 (31.0)
40s	458 (27.8)	118 (26.8)	81 (26.2)	259 (28.8)
50 or older	358 (21.7)	139 (31.5)	78 (25.2)	141 (15.7)
Educational background ^3^				
Vocational school	72 (4.4)	15 (3.4)	23 (7.4)	34 (3.8)
Junior college	294 (17.8)	43 (9.8)	44 (14.2)	207 (23.0)
Advanced course in junior college	48 (2.9)	9 (2.0)	6 (1.9)	33 (3.7)
University	1145 (69.4)	334 (75.7)	210 (68.0)	601 (66.9)
Master’s course in graduate school	87 (5.3)	39 (8.8)	25 (8.1)	23 (2.6)
Doctor’s course in graduate school	3 (0.2)	1 (0.2)	1 (0.3)	1 (0.1)
Current position				
Officer	1407 (85.3)	388 (88.0)	255 (82.5)	764 (85.0)
Supervisor/management	242 (14.7)	53 (12.0)	54 (17.5)	135 (15.0)

*n* (%). ^1^ Designated cities. ^2^ Excluding designated cities. ^3^ Registered dietitian/dietitian certification or related field.

**Table 2:** Association between years of experience in health promotion and individual learning needs in prefectures.

	Total (*n* = 441)	Early ^1^ (*n* = 202)	Mid-Career ^2^ (*n* = 125)	Leadership ^3^ (*n* = 114)
The desired final position is a supervisor/management	235 (53.3)	106 (52.5) 1 (ref.)	65 (52.0) 0.98 (0.63–1.53)	64 (56.1) 1.16 (0.73–1.84)
Intended career path ^4^				
I want to continue working as a nutrition specialist.	192 (43.5)	100 (49.5) 1 (ref.)	48 (38.4) 0.64 (0.40–1.00)	44 (38.6) 0.64 (0.40–1.02)
I want to be promoted and work as a nutrition specialist.	95 (21.5)	44 (21.8) 1 (ref.)	25 (20.0) 0.90 (0.52–1.56)	26 (22.8) 1.06 (0.61–1.84)
I want to continue working as a public health generalist.	116 (26.3)	38 (18.8) 1 (ref.)	35 (28.0) 1.68 (0.99–2.84)	43 (37.7) 2.61 (1.56–4.39)
I want to be promoted and work as a public health generalist.	99 (22.4)	38 (18.8) 1 (ref.)	29 (23.2) 1.30 (0.76–2.25)	32 (28.1) 1.68 (0.98–2.89)
I want to transfer to a department outside of health promotion services (in a bureau).	99 (22.4)	44 (21.8) 1 (ref.)	41 (32.8) 1.75 (1.06–2.89)	14 (12.3) 0.50 (0.26–0.96)
I want to transfer to a department (outside bureau such as a hospital).	73 (16.6)	46 (22.8) 1 (ref.)	20 (16.0) 0.65 (0.36–1.15)	7 (6.1) 0.22 (0.10–0.51)
I want to return to a department outside of health promotion where I previously worked.	13 (2.9)	5 (2.5) 1 (ref.)	4 (3.2) 1.30 (0.34–4.95)	4 (3.5) 1.43 (0.38–5.45)
I want to change my job type (e.g., clerical work).	19 (4.3)	9 (4.5) 1 (ref.)	8 (6.4) 1.47 (0.55–3.91)	2 (1.8) 0.38 (0.08–1.80)
I want to move to a ministry or other government agency.	12 (2.7)	10 (5.0) 1 (ref.)	2 (1.6) 0.31 (0.67–1.45)	0 (0.0) –
I want to change jobs outside of government (education/research).	26 (5.9)	14 (6.9) 1 (ref.)	4 (3.2) 0.44 (0.14–1.38)	8 (0.7) 1.01 (0.41–2.49)
I want to change jobs outside of government (non-education/research).	42 (9.5)	26 (12.9) 1 (ref.)	11 (8.8) 0.65 (0.31–1.37)	5 (4.4) 0.31 (0.12–0.83)
I want to retire (I have no desire to work).	18 (4.1)	3 (1.5) 1 (ref.)	5 (4.0) 2.76 (0.65–11.8)	10 (8.8) 6.38 (1.72–23.7)
Skills that the participants felt they needed to improve in the future ^4^				
Administrative competence (e.g., professional ethics, code of conduct)	74 (16.8)	33 (16.3) 1 (ref.)	23 (18.4) 1.15 (0.64–2.08)	18 (15.8) 0.96 (0.51–1.80)
Professional competence (e.g., knowledge in specialized areas, nutritional guidance techniques)	169 (38.3)	114 (56.4) 1 (ref.)	30 (24.0) 0.24 (0.15–0.40)	25 (21.9) 0.22 (0.13–0.37)
Community support capacity (e.g., interprofessional work, work with residents)	120 (27.2)	64 (31.7) 1 (ref.)	32 (25.6) 0.74 (0.45–1.22)	24 (21.1) 0.58 (0.34–0.99)
Research competence (e.g., information gathering, data analysis, reporting practical solutions)	167 (37.9)	83 (41.1) 1 (ref.)	47 (37.6) 0.86 (0.55–1.37)	37 (32.5) 0.69 (0.43–1.12)
Coordination competence (e.g., leadership, consensus building, logical explanations, communications)	204 (46.3)	81 (40.1) 1 (ref.)	61 (48.8) 1.42 (0.91–2.23)	62 (54.4) 1.78 (1.12–2.83)
Policy-making competence (e.g., policy making and evaluations based on the PDCA cycle).	176 (39.9)	81 (40.1) 1 (ref.)	49 (39.2) 0.96 (0.61–1.52)	46 (40.4) 1.01 (0.63–1.61)
Information dissemination competence (e.g., presentations, work visualization).	142 (32.2)	70 (34.7) 1 (ref.)	35 (28.0) 0.73 (0.45–1.19)	37 (32.5) 0.91 (0.56–1.48)
Organizational management competence (e.g., appropriate acquisition of “manpower, materials, and capital”).	99 (22.4)	23 (11.4) 1 (ref.)	42 (33.6) 3.94 (2.22–6.97)	34 (29.8) 3.31 (1.83–5.97)
Risk management competence (e.g., response to disasters, food poisoning, infectious diseases, etc.)	112 (25.4)	39 (19.3) 1 (ref.)	40 (32.0) 1.97 (1.18–3.29)	33 (28.9) 1.70 (1.00–2.91)
Other	5 (1.1)	1 (0.5) 1 (ref.)	0 (0.0) –	4 (3.5) 7.31 (0.81–66.2)

*n* (%) or odds ratio (95% confidence interval) in binary logistic regression analysis. ^1^ <10 years. ^2^ 10–19 years. ^3^ ≥20 years. ^4^ Participants were asked to respond to up to three items they felt were particularly applicable.

**Table 3:** Association between years of experience in health promotion and individual learning needs in cities ^1^.

	Total (*n* = 309)	Early ^2^ (*n* = 174)	Mid-Career ^3^ (*n* = 82)	Leadership ^4^ (*n* = 53)
The desired final position is a supervisor/management	150 (48.5)	81 (46.6) 1 (ref.)	40 (48.8) 1.09 (0.65–1.85)	29 (54.7) 1.39 (0.75–2.57)
Intended career path ^5^				
I want to continue working as a nutrition specialist.	185 (59.9)	107 (61.5) 1 (ref.)	51 (62.2) 1.03 (0.60–1.77)	27 (50.9) 0.65 (0.35–1.21)
I want to be promoted and work as a nutrition specialist.	57 (18.4)	34 (19.5) 1 (ref.)	14 (17.1) 0.85 (0.43–1.68)	9 (17.0) 0.84 (0.38–1.89)
I want to continue working as a public health generalist.	54 (17.5)	27 (15.5) 1 (ref.)	17 (20.7) 1.42 (0.73–2.79)	10 (18.9) 1.27 (0.57–2.82)
I want to be promoted and work as a public health generalist.	38 (12.3)	14 (8.0) 1 (ref.)	14 (17.1) 2.35 (1.06–5.20)	10 (18.9) 1.46 (0.53–4.01)
I want to transfer to a department outside of health promotion services (in a bureau).	60 (19.4)	30 (17.2) 1 (ref.)	17 (20.7) 1.26 (0.65–2.44)	13 (24.5) 1.56 (0.74–3.27)
I want to transfer to a department (outside bureau such as a hospital).	24 (7.8)	17 (9.8) 1 (ref.)	4 (4.9) 0.47 (0.15–1.46)	3 (5.7) 0.55 (0.16–1.97)
I want to return to a department outside of health promotion where I previously worked.	5 (1.6)	5 (2.9) 1 (ref.)	0 (0.0) –	0 (0.0) –
I want to change my job type (e.g., clerical work).	16 (5.2)	9 (5.2) 1 (ref.)	4 (4.9) 0.94 (0.28–3.15)	3 (5.7) 1.10 (0.29–4.22)
I want to move to a ministry or other government agency.	15 (4.9)	11 (6.3) 1 (ref.)	2 (2.4) 0.37 (0.08–1.71)	2 (3.8) 0.58 (0.12–2.71)
I want to change jobs outside of government (education/research).	22 (7.1)	14 (8.0) 1 (ref.)	5 (6.1) 0.74 (0.26–2.13)	3 (5.7) 0.69 (0.19–2.48)
I want to change jobs outside of government (non-education/research).	30 (9.7)	19 (10.9) 1 (ref.)	7 (8.5) 0.76 (0.31–1.89)	4 (7.5) 0.67 (0.22–2.05)
I want to retire (I have no desire to work).	9 (2.9)	4 (2.3) 1 (ref.)	2 (2.4) 1.06 (0.19–5.92)	3 (5.7) 2.55 (0.55–11.8)
Skills that the participants felt they needed to improve in the future ^5^				
Administrative competence (e.g., professional ethics, code of conduct)	57 (18.4)	38 (21.8) 1 (ref.)	11 (13.4) 0.55 (0.27–1.15)	8 (15.1) 0.64 (0.28–1.46)
Professional competence (e.g., knowledge in specialized areas, nutritional guidance techniques)	175 (56.6)	123 (70.7) 1 (ref.)	31 (37.8) 0.25 (0.14–0.44)	21 (39.6) 0.27 (0.14–0.52)
Community support capacity (e.g., interprofessional work, work with residents)	96 (31.1)	61 (35.1) 1 (ref.)	21 (25.6) 0.64 (0.36–1.15)	14 (26.4) 0.66 (0.34–1.32)
Research competence (e.g., information gathering, data analysis, reporting practical solutions)	99 (32.0)	57 (32.8) 1 (ref.)	25 (30.5) 0.90 (0.51–1.59)	17 (32.1) 0.97 (0.50–1.87)
Coordination competence (e.g., leadership, consensus building, logical explanations, communications)	126 (40.8)	69 (39.7) 1 (ref.)	36 (43.9) 1.19 (0.70–2.03)	21 (39.6) 1.00 (0.53–1.87)
Policy-making competence (e.g., policy making and evaluations based on the PDCA cycle).	97 (31.4)	45 (25.9) 1 (ref.)	31 (37.8) 1.74 (0.99–3.05)	21 (39.6) 1.88 (0.98–3.59)
Information dissemination competence (e.g., presentations, work visualization).	102 (33.0)	49 (28.2) 1 (ref.)	37 (45.1) 2.10 (1.21–3.62)	16 (30.2) 1.10 (0.56–2.16)
Organizational management competence (e.g., appropriate acquisition of “manpower, materials, and capital”).	62 (20.1)	29 (16.7) 1 (ref.)	22 (26.8) 1.83 (0.98–3.44)	11 (20.8) 1.31 (0.60–2.84)
Risk management competence (e.g., response to disasters, food poisoning, infectious diseases, etc.)	70 (22.7)	32 (18.4) 1 (ref.)	23 (28.0) 1.73 (0.93–3.20)	15 (28.3) 1.75 (0.86–3.56)
Other	3 (1.0)	1 (0.6) 1 (ref.)	1 (1.2) 2.14 (0.13–34.6)	1 (1.9) 3.33 (0.20–54.1)

*n* (%) or odds ratio (95% confidence interval) in binary logistic regression analysis. ^1^ Designated cities. ^2^ <10 years. ^3^ 10–19 years. ^4^ ≥20 years. ^5^ Participants were asked to respond to up to three items they felt were particularly applicable.

**Table 4:** Association between years of experience in health promotion and individual learning needs in municipalities ^1^.

	Total (*n* = 899)	Early ^2^ (*n* = 600)	Mid-Career ^3^ (*n* = 183)	Leadership ^4^ (*n* = 116)
The desired final position is a supervisor/management	448 (49.8)	265 (44.2) 1 (ref.)	103 (56.3) 1.63 (1.17–2.27)	80 (69.0) 2.81 (1.84–4.30)
Intended career path ^5^				
I want to continue working as a nutrition specialist.	608 (67.6)	397 (66.2) 1 (ref.)	129 (70.5) 1.22 (0.85–1.75)	82 (70.7) 1.23 (0.80–1.90)
I want to be promoted and work as a nutrition specialist.	159 (17.7)	117 (19.5) 1 (ref.)	29 (15.8) 0.78 (0.50–1.21)	13 (11.2) 0.52 (0.28–0.96)
I want to continue working as a public health generalist.	156 (17.4)	86 (14.3) 1 (ref.)	33 (18.0) 1.31 (0.85–2.04)	37 (31.9) 2.80 (1.78–4.40)
I want to be promoted and work as a public health generalist.	77 (8.6)	46 (7.7) 1 (ref.)	24 (13.1) 1.82 (1.08–3.07)	7 (6.0) 0.77 (0.34–1.76)
I want to transfer to a department outside of health promotion services (in a bureau).	128 (14.2)	86 (14.3) 1 (ref.)	31 (16.9) 1.22 (0.78–1.91)	11 (9.5) 0.63 (0.32–1.21)
I want to transfer to a department (outside bureau such as a hospital).	41 (4.6)	28 (4.7) 1 (ref.)	7 (3.8) 0.81 (0.35–1.89)	6 (5.2) 1.11 (0.45–2.75)
I want to return to a department outside of health promotion where I previously worked.	12 (1.3)	8 (1.3) 1 (ref.)	2 (1.1) 0.82 (0.17–3.88)	2 (1.7) 1.30 (0.27–6.19)
I want to change my job type (e.g., clerical work).	45 (5.0)	32 (5.3) 1 (ref.)	10 (5.5) 1.03 (0.49–2.13)	3 (2.6) 0.47 (0.14–1.57)
I want to move to a ministry or other government agency.	28 (3.1)	26 (4.3) 1 (ref.)	1 (0.5) 0.12 (0.02–0.90)	1 (0.9) 0.19 (0.03–1.43)
I want to change jobs outside of government (education/research).	43 (4.8)	31 (5.2) 1 (ref.)	10 (5.5) 1.06 (0.51–2.21)	2 (1.7) 0.32 (0.08–1.36)
I want to change jobs outside of government (non-education/research).	104 (11.6)	74 (12.3) 1 (ref.)	20 (10.9) 0.87 (0.52–1.47)	10 (8.6) 0.67 (0.34–1.34)
I want to retire (I have no desire to work).	36 (4.0)	18 (3.0) 1 (ref.)	7 (3.8) 1.29 (0.53–3.13)	11 (9.5) 3.39 (1.56–7.38)
Skills that the participants felt they needed to improve in the future ^5^				
Administrative competence (e.g., professional ethics, code of conduct)	211 (23.5)	148 (24.7) 1 (ref.)	37 (20.2) 0.77 (0.52–1.16)	26 (22.4) 0.88 (0.55–1.42)
Professional competence (e.g., knowledge in specialized areas, nutritional guidance techniques)	642 (71.4)	475 (79.2) 1 (ref.)	103 (56.3) 0.34 (0.24–0.48)	64 (55.2) 0.32 (0.21–0.49)
Community support capacity (e.g., interprofessional work, work with residents)	379 (42.2)	263 (43.8) 1 (ref.)	59 (32.2) 0.61 (0.43–0.86)	57 (49.1) 1.24 (0.83–1.84)
Research competence (e.g., information gathering, data analysis, reporting practical solutions)	240 (26.7)	155 (25.8) 1 (ref.)	55 (30.1) 1.23 (0.86–1.78)	30 (25.9) 1.00 (0.64–1.58)
Coordination competence (e.g., leadership, consensus building, logical explanations, communications)	289 (32.1)	184 (30.7) 1 (ref.)	64 (35.0) 1.22 (0.86–1.73)	41 (35.3) 1.24 (0.81–1.88)
Policy-making competence (e.g., policy making and evaluations based on the PDCA cycle).	245 (27.3)	140 (23.3) 1 (ref.)	69 (37.7) 1.99 (1.40–2.83)	36 (31.0) 1.48 (0.96–2.29)
Information dissemination competence (e.g., presentations, work visualization).	251 (27.9)	163 (27.2) 1 (ref.)	62 (33.9) 1.37 (0.96–1.96)	26 (22.4) 0.77 (0.48–1.24)
Organizational management competence (e.g., appropriate acquisition of “manpower, materials, and capital”).	144 (16.0)	74 (12.3) 1 (ref.)	39 (21.3) 1.93 (1.25–2.96)	31 (26.7) 2.59 (1.61–4.18)
Risk management competence (e.g., response to disasters, food poisoning, infectious diseases, etc.)	147 (16.4)	96 (16.0) 1 (ref.)	29 (15.8) 0.99 (0.63–1.55)	22 (19.0) 1.23 (0.74–2.05)
Other	11 (1.2)	8 (1.3) 1 (ref.)	2 (1.1) 0.82 (0.17–3.88)	1 (0.9) 0.64 (0.08–5.19)

*n* (%) or odds ratio (95% confidence interval) in binary logistic regression analysis. ^1^ Excluding designated cities. ^2^ <10 years. ^3^ 10–19 years. ^4^ ≥20 years. ^5^ Participants were asked to respond to up to three items they felt were particularly applicable.

## Data Availability

The datasets generated and analyzed during the current study are not publicly available due to privacy and ethical restrictions but are available from the corresponding author on reasonable request.
